# Mental Activity as the Bridge between Neural Biomarkers and Symptoms of Psychiatric Illness

**DOI:** 10.1177/15500594221112417

**Published:** 2022-07-21

**Authors:** Justin Riddle, Flavio Frohlich

**Affiliations:** 1 Department of Psychiatry, 6797 University of North Carolina at Chapel Hill, Chapel Hill, NC, USA; 2 Carolina Center for Neurostimulation, 6797 University of North Carolina at Chapel Hill, Chapel Hill, NC, USA; 3 Department of Neurology, 6797 University of North Carolina at Chapel Hill, Chapel Hill, NC, USA; 4 Department of Cell Biology and Physiology, 6797 University of North Carolina at Chapel Hill, Chapel Hill, NC, USA; 5 Department of Biomedical Engineering, 6797 University of North Carolina at Chapel Hill, Chapel Hill, NC, USA; 6 Neuroscience Center, 6797 University of North Carolina at Chapel Hill, Chapel Hill, NC, USA

**Keywords:** causal validation, dimensionality reduction, neurobehavioral model, RDoC, symptom dimensions

## Abstract

The Research Domain Criteria (RDoC) initiative challenges researchers to build neurobehavioral models of psychiatric illness with the hope that such models identify better targets that will yield more effective treatment. However, a guide for building such models was not provided and symptom heterogeneity within Diagnostic Statistical Manual categories has hampered progress in identifying endophenotypes that underlie mental illness. We propose that the best chance to discover viable biomarkers and treatment targets for psychiatric illness is to investigate a triangle of relationships: severity of a specific psychiatric symptom that correlates to mental activity that correlates to a neural activity signature. We propose that this is the minimal model complexity required to advance the field of psychiatry. With an understanding of how neural activity relates to the experience of the patient, a genuine understanding for how treatment imparts its therapeutic effect is possible. After the discovery of this three-fold relationship, causal testing is required in which the neural activity pattern is directly enhanced or suppressed to provide causal, instead of just correlational, evidence for the biomarker. We suggest using non-invasive brain stimulation (NIBS) as these techniques provide tools to precisely manipulate spatial and temporal activity patterns. We detail how this approach enabled the discovery of two orthogonal electroencephalography (EEG) activity patterns associated with anhedonia and anxiosomatic symptoms in depression that can serve as future treatment targets. Altogether, we propose a systematic approach for building neurobehavioral models for dimensional psychiatry.

## Minimal Complexity for a Neurobehavioral Psychiatric Model

Patients with psychiatric illness often present with psychiatric comorbidities and heterogenous symptom expression.^1–3^ For example, two patients diagnosed with major depressive disorder (MDD) under the Diagnostic Statistical Manual version-5 (DSM-V) could present with entirely non-overlapping symptoms. However, first-line treatment is often identical despite differences in symptoms; and the journey towards optimal treatment can be a long trial-and-error process.^4,5^ A better understanding of the neural and behavioral systems that underlie distinct symptoms of psychiatric illness could save critical time in reaching optimal treatment. Furthermore, causally validated neurobehavioral models are essential to guide the development of evidence-based personalized treatment, eg, non-invasive brain stimulation (NIBS) targeted directly to neural activity or psychotherapy directed at the underlying mental activity.

**Figure 1.**
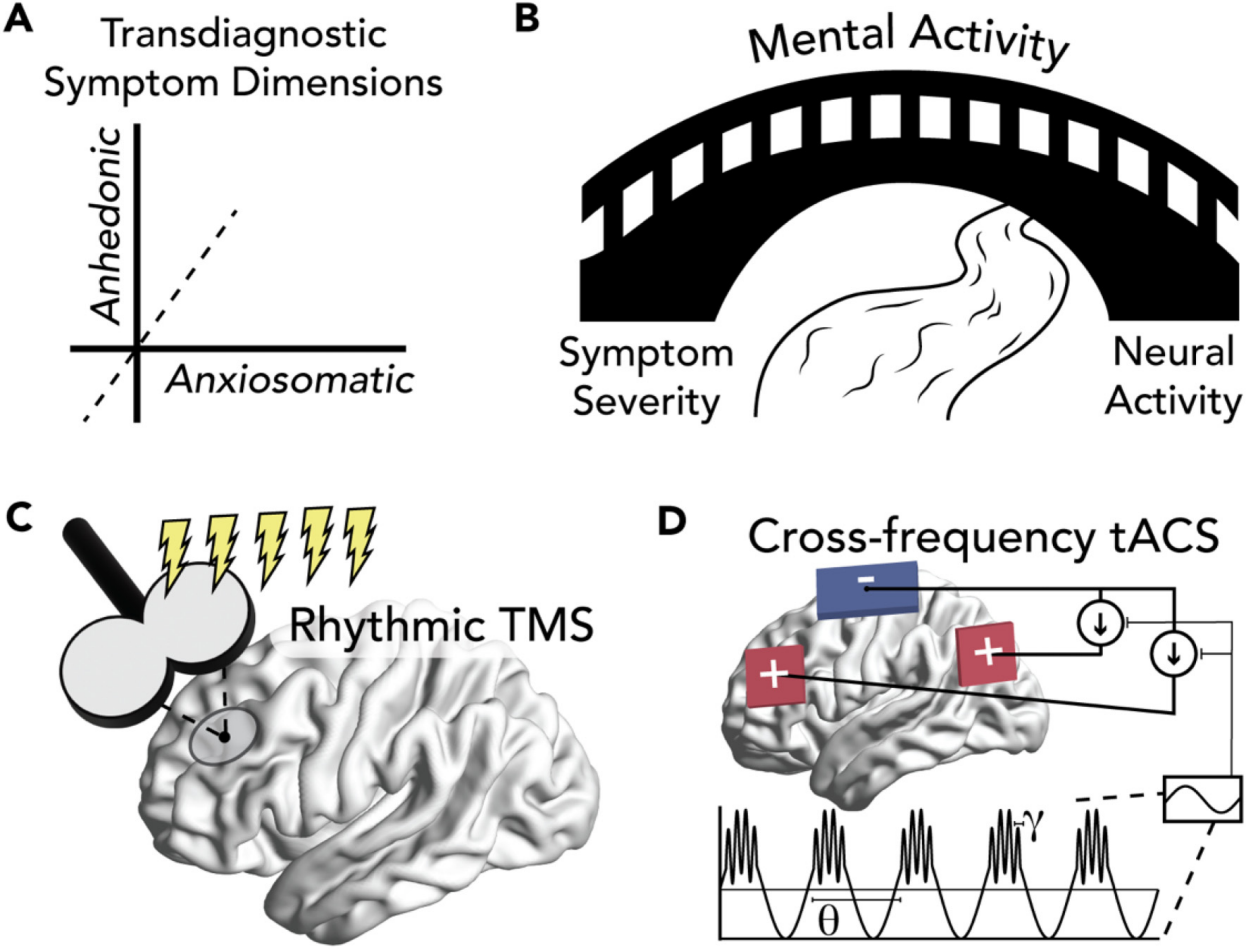
Mental activity as the bridge between biomarkers and symptom of psychiatric illness. (A) Psychiatric illness comprises symptoms that are transdiagnostic. Symptom heterogeneity within a diagnostic category can be investigated using dimensionality reduction, eg, anhedonic and anxiosomatic symptoms within major depressive disorder. (B) Mental activity is the bridge between symptom severity and neural activity. After identifying a psychiatric symptom, models that account for the experience of the patient will yield biomarkers with greater explanatory power. Causal testing of these models is necessary to move beyond correlational evidence, eg, (C) rhythmic transcranial magnetic stimulation (TMS) offers precise spatial targeting and (D) cross-frequency transcranial alternating current stimulation (tACS) offers precise temporal targeting.

Diagnostic categories are a useful starting point for understanding psychiatric illness; but with widespread comorbidity between categories and extensive specification under an umbrella category, a new approach grounded in biology appears necessary. The National Institute of Mental Health affirmed this observation by emphasizing that: an understanding of mental activity is fundamental to advancing understanding of psychiatric illness and the development of new treatments. With the Research Domain Criteria (RDoC), the research community was encouraged to integrate advances from cognitive neuroscience^6^ into research of novel biomarkers for psychiatric illness. Given the complexity of this undertaking, no guide was provided to deconstruct psychiatric illness into symptom dimensions (Figure 1A) related to mental activity and implemented with a neural mechanism. Here, we attempt to address this gap by demonstrating how an interdisciplinary approach that focuses on synergistic integration of the fields of psychiatry research, network neuroscience, and cognitive science can provide novel insights that are actionable in terms of treatment development. Critically, we propose that mental activity, defined as higher-order brain-based processes that instantiate the complex connections between sensory input and motor output, is the bridge between psychiatric symptoms and neural activity biomarkers (Figure 1B). In addition, we emphasize the importance of causal testing to advance a neurobehavioral model beyond correlation and describe recent advances in using rhythmic transcranial magnetic stimulation (TMS) (Figure 1C) and cross-frequency transcranial alternating current stimulation (tACS) (Figure 1D).

## Dimension Discovery or Focusing on a Symptom

The overarching categories of psychiatric illness carve the expanse of human experience of psychiatric illness into tractable points of focus. While this labeling process is a useful first step in the clinic where treatment (ideally) becomes progressively individualized as the clinician becomes familiar with the client, these categories have proven to be inadequate in establishing a neural basis for psychiatric illness.^7,8^ There will never be a “depression” or a “schizophrenia” region of the brain just as there will never be a “love” or “hope” circuit. In the field of cognitive neuroscience, researchers discovered that in order to find robust neural correlates, mental activity needs to be described in simple and generalizable terms.^9^ Then, the contexts by which that mental activity is generated can be explored with concurrent neuroimaging or electrophysiology. For example, selective attention is the allocation of limited resources to one stimulus at the expense of another^10^ and working memory is the maintenance of information over a brief period of time through active intention.^11^ Similarly, RDoC requests that the conceptualization of psychiatric illness be understood as changes in fundamental constructs, eg, cognitive control, reward learning, and emotion regulation, that are transdiagnostic and embedded in multiscale neurobiology. In our opinion, the best candidate framework that leverages existing literature and relates psychiatric illness to fundamental constructs is a symptom-focused approach.^12^ A symptom is more amenable to a neural description because it avoids the heterogeneity of broad diagnostic categories. Researchers can focus on a single symptom or characterize a psychiatric illness as a confluence of multiple symptom dimensions. Each symptom dimension can be conceptualized as a continuum from “no symptom expression” to “severe symptom expression.”

While the symptom-dimension approach may provide novel biomarkers beyond categorical approaches, researchers should continue to use diagnostic tools, eg, Structured Clinical Interview for DSM. Diagnosis increases interpretability within the field of psychiatry and allows for a comparison of the dimensional versus traditional approach. In a recent publication, we modeled MDD as a composition of two symptom dimensions derived from our data.^13^ One dimension encompassed symptoms of anhedonia, reduced motivation, and melancholia and the second dimension encompassed symptoms of anxiety, rumination, and depressed mood. Even though our dimensionality reduction included only 38 patients, we reproduced similar symptom dimensions found by other groups using datasets with over 1000 patients.^14,15^ These symptom dimensions yielded relationships to behavior and neural activity not present at the diagnostic category level.

The dimensional approach enables investigation of a transdiagnostic patient population with a common symptom. An ideal candidate for research is two diagnostic categories that are often found to be comorbid and investigation can be focused on common symptoms, eg, rumination in anxiety disorders and MDD^16,17^ or anhedonia in depression and schizophrenia.^18^ The common symptoms could be selected a priori or discovered through dimensionality reduction. Consideration should be paid to which assessments are included in the experiment and how dimensionality reduction is performed. If the experiment is limited in the number of participants that can be recruited (eg, an expensive fMRI experiment), then we recommend that experimenters rely on previously established symptom dimensions and employ existing assessments. Alternatively, a symptom-dimension discovery phase can be conducted using a larger participant base and a battery of clinical assessments.

A clinical assessment battery should sufficiently sample the space of possible symptoms. We recommend using established clinical assessments with known psychometric properties, although theoretically experimenters could develop assessments with subscales targeted to hypothesized or discovered symptom dimensions. However, concerns about the validity of ad-hoc scales need to be taken seriously and rigorous assay development techniques must be employed. In our recent experiment, we administered many assessments relevant to theorized dimensions of depression: an anhedonia and anxiety/mood-dysregulation dimension. We included standard anhedonia assessments, Snaith-Hamilton Pleasure Scale^19^ and Temporal Experience of Pleasure Scale (consummatory and anticipatory subscale),^20^ and an assessment to capture behavioral approach: the Behavioral Activation Scale^21^ with subscales for drive, fun-seeking, reward-responsiveness and the Positive Affect Schedule.^22^ As expected, these scales were highly correlated with each other. In addition, we probed anxiety, rumination, and depressed mood using a wide range of scales: State-Trait Anxiety Inventory,^23^ Ruminative Response Scale,^24^ Behavioral Inhibition Scale,^21^ and Negative Affect Schedule.^22^ As hypothesized, these scales were highly correlated with each other across individuals. By using multiple assessments to probe aspects of a symptom profile, the experimenter can validate the consistency of the underlying construct and avoid biases inherent to any individual assessment.

In addition, we included general depression scales. However, these scales conflate symptom dimensions in an attempt to quantify severity within the overarching diagnostic category. For this reason, the Beck's Depression Inventory version-2^25^ and the Hamilton Depression Rating Scale (17-item)^26^ should not be used in isolation in dimension-discovery analyzes. In our experiment, we ran a dimension-discovery analysis on the individual items of these assessments using prior research for validation: independent groups have found separable symptom dimensions within the HAM-D and the BDI-II.^14,27^ In our data, we replicated these dimensions and submitted these subscales to the primary dimension discovery analysis.

Dimension-discovery is inevitably driven by hypotheses, because an initial decision of what assessments to include is necessary. As a general recommendation, experimenters should use multiple assessments to triangulate the hypothesized symptoms and include a roughly matched number of subscales per symptom of interest, eg, roughly 8 in our experiment. We recommend that dimension-discovery begins with relatively few hypothesized dimensions. Our analysis hypothesized the presence of two dimensions, which was feasible with 38 participants in a depressive episode. While future models might expand this model to include a third or perhaps fourth dimension, we expect that the explanatory power and generalization of higher dimensional models will rapidly decrease and be susceptible to overfitting.

**Figure 2.**
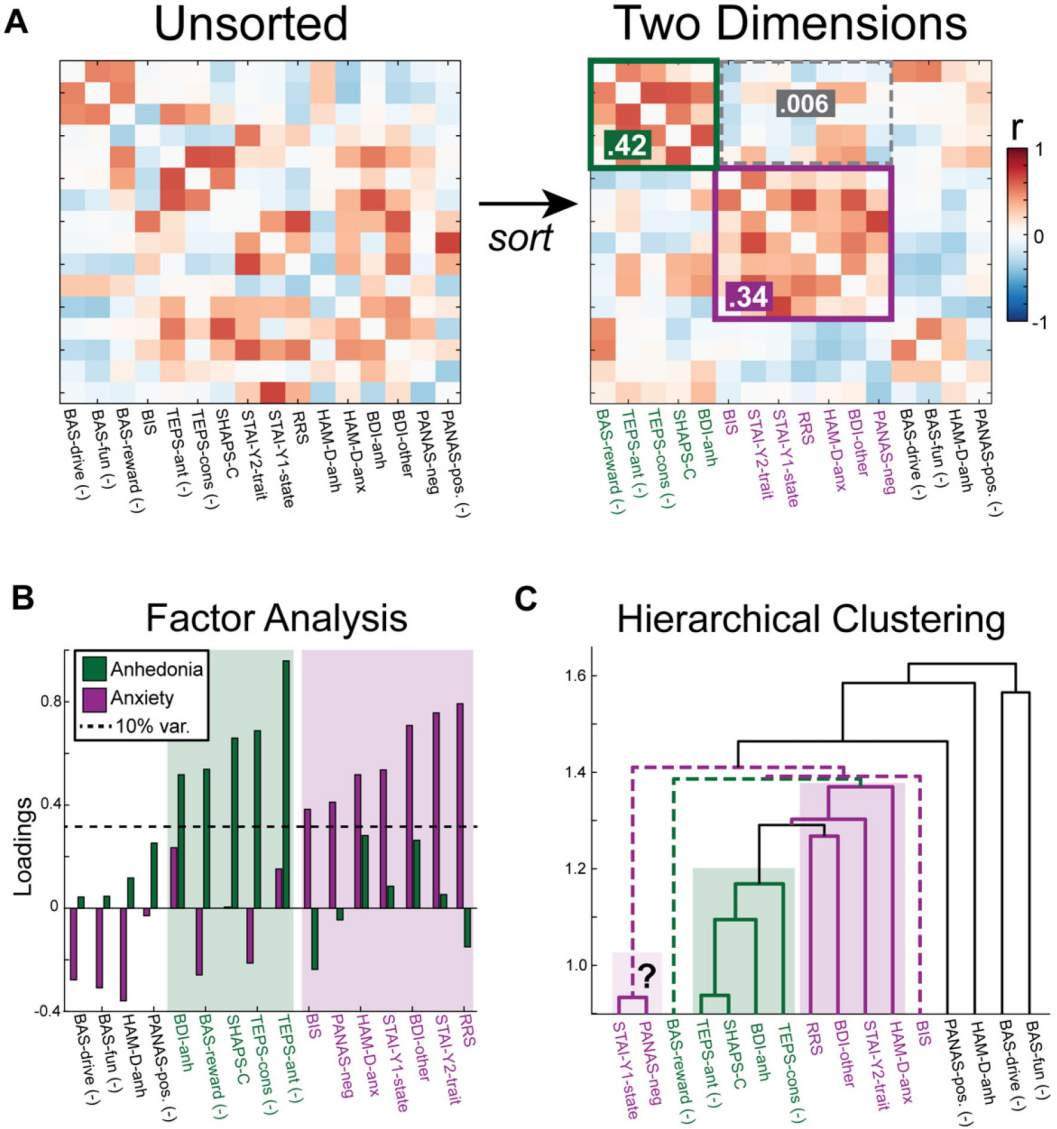
Dimensionality reduction for classifying psychiatric symptoms. (A) The goal of dimension discovery is to translate an unsorted covariance matrix into distinct clusters with a high internal correlation and low inter-dimensional correlation. The values are the average within-dimension (green and purple) and between-dimension correlation values (grey). Two prominent methods for conducting dimension discovery are (B) factor analysis and (C) hierarchical clustering. (B) For factor analysis, the a priori threshold of 10% explained variance (dashed line) is used to define the inclusion of a subscale in the dimension. The anhedonia dimension is labeled in green and the anxiety dimension is labeled in purple. (C) Hierarchical clustering yields smaller clusters (solid lines) and it is unclear how to interpret higher order associations (dashed lines). Coloring based on factor analysis.

After selecting clinical assessments, a dimensionality reduction technique separates distinct dimensions. Successful dimensionality reduction is apparent when the covariance matrix is sorted by symptom assignment to yield clear clusters with strong intradimensional and weak interdimensional correlation (Figure 2A). These techniques either impose a predetermined set of dimensions such as factor analysis (Figure 2B), or flexibly allow for dimension assignment such as hierarchical clustering (Figure 2C). Both methods require a technique for establishing symptom dimensions. Previous research suggests that a threshold of 10% explained variance (factor loading of 0.33) serves as an a priori threshold for including the subscale in the symptom composite.^28^ If a subscale shows a significant loading for multiple dimensions, then we recommend exclusion as the subscale likely conflates the dimensions of interest.

Establishing dimensions in hierarchical clustering is less straightforward. The hierarchical clustering algorithm is iterative: subscales with the strongest correlation are grouped first. Clustering often yields one strongly interconnected dimension (anhedonia cluster in Figure 2C), small groupings of two or three subscales with high correlation, and an unclear clustering-structure beyond the lowest levels. There is no a priori similarity value to establish a cluster nor a guarantee of finding multiple clusters.

After identifying the subscales to be included in each dimension, each subscale should be normalized and averaged. We recommend a z-transformation across the distribution of all participants that will be included in the analysis. Then, before relating symptom severity to mental activity, the composite symptom dimensions should be visualized in multidimensional space. If the data reveal an outlier cluster, then there are likely non-linearities in the symptom dimensions. For example, after establishing symptom dimensions, one group opted to cluster participants into “subtypes” of depression.^14^ Then, future analyzes were run as a difference between “subtypes.” In our analysis, we localized symptom dimensions in participants in a major depressive episode, but ran our analysis across a wider pool of participants that included euthymic controls. Thus, our symptom dimensions were correlated with each other in the larger group. Statistical analysis addressed this relationship by including both symptom dimensions in a linear model and by using post-hoc partial correlation analyses that accounted for variance in the other symptom dimension.

## Mental Activity during Symptom Expression

Similar to the vast space of all possible psychiatric illnesses, the complexity of human thought is proposed to arise from the confluence of distinct mental activities, referred to as cognitive processes in the field of cognitive neuroscience.^9,29^ We use the term mental activity here, as the field of psychiatry often uses the term “cognitive” to exclude emotional and affective processes. Mental activity comprises the processing of stimuli in the environment, manipulating internally-generated representations, and orienting behavior towards abstract goals. We propose that symptoms of psychiatric illness will display a characteristic alteration in certain mental activities.^13^ The experience of a psychiatric symptom is likely *isomorphic* with an under-expression, overexpression, or alteration of some mental activity. Of concern, most investigations that attempt to find a biomarker for psychiatric illness treat the brain as a static system and acquire functional magnetic resonance imaging (fMRI) or electroencephalography (EEG) data during the resting-state. The resting-state is a dynamical state with many mental activities and associated brain networks, activating and switching from one to another.^30^ Instead of quantifying and tracking these different forms of mental activity, researchers expect that with a long recording, trait-like forms of mental activity will emerge from the temporal average. While these investigations uncovered some biomarkers, these biomarkers often do not replicate across repeated samples, eg, frontal alpha asymmetry in MDD,^7^ and explanatory power is limited as the mental activity of the patient is unknown.

We propose that active or passive tasks be designed to generate an acute expression of the target symptom such that the associated mental can be studied, eg, passively viewing emotionally charged images^31^ or performing a reward-based decision-making task.^13^ Alternatively, a task may drive the circuit that mediates the symptom by eliciting the associated mental activity allowing individual difference to be analyzed, but may not result in an expression of the symptom in the patient. Future research should pay mind to what degree the symptom to mental activity relation is direct (the former) or indirect (the latter). We propose that more robust biomarkers for symptoms of psychiatric illness are revealed in the context-dependent state.

For example, in our recent experiment, participants with MDD performed a reward-based decision-making task designed to probe two mental activities, goal-directed behavior and reward-evaluation, known to be altered in MDD.^1,13^ We found a negative relationship between symptoms of anhedonia and goal-directed behavior, and a positive relationship between anxiety and reward-evaluation. Thus, symptom dimensions of MDD may alter behavior through anomalies in distinct mental activities. Consistent with previous research, symptoms of anhedonia, the reduced experience of pleasure from leisure activities, is related to a deeper construct of decreased motivation to direct behavior towards abstract goals when physical effort is required.^1,32^ The inability to experience pleasure may stem from reduced time spent actively seeking experiences that will confer pleasure. Intriguingly, this relationship is not intuitive from the language of the clinical assessments alone; the statement “I find pleasure in my hobbies and pastimes” does not intuitively suggest a deficit in motivation to seek out rewarding experiences. Here, we gained insight into the experience of the patient and derived a behavioral metric for quantifying the severity of alteration in this mental activity. However, the investigator should be careful to consider whether a more fundamental construct exists such that the investigated relationship between symptom and mental activity is emergent.

By comparison, anxiety symptoms corresponded with increased strategic allocation of effort to maximize reward acquisition. Thus, anxiety was not detrimental to performance within the context of the task. This suggests that while our task was able to capture variability in the mental activity associated with anxiety, the reward-based decision-making task used here was unable to reveal its pathology: intriguingly, anxiety was associated with increased cognitive control. Future work is required to further specify under what condition this tendency to exert control becomes distressing and results in decreased performance. Perhaps anxiety increases the rate at which cognitive resources are expended leading to a break in control when the participant is overwhelmed, or anxious rumination could lead to an overemphasis of punishment and a deficit in reward learning from positive reinforcement.^33^

The choice of task should be motivated by the potential to explain a symptom dimension in its most simple context. Considerations for effective experimental design are covered in other reviews,^34^ so we will focus on considerations specific to dimensional psychiatry. Similar to selecting a battery of clinical assessments, a battery of cognitive tasks can be used to triangulate relevant mental activities that are hypothesized to be altered by the psychiatric symptoms of interest. Ideally, a different mental activity is implicated in each symptom dimension; but an experiment may also identify a single mental activity that is altered along one symptom dimension and not another. Given the time required to collect behavioral data in a task, we recommend using either two different tasks with a behavioral metric hypothesized to be related to each mental activity or a single task that drives multiple mental activities.

A critical decision for task design is behavioral titration, the method by which task performance is ensured to neither be “at ceiling,” near perfect performance, nor “at floor,” near chance level with poor performance. If performance is at floor, then the participant is not engaged in the mental activity under investigation. If performance is at ceiling, then correlation analysis between behavior and symptom severity will fail from a lack of interindividual variability. The task should include a behavioral titration phase where task difficulty is adjusted such that performance is an optimal range for each participant. This concern does not apply to clinical assessments, which are designed to capture interindividual variability.

There is no simple statistical model to relate two independent variables (eg, severity of two symptoms) to two dependent variables (eg, two behavioral metrics). We recommend running a linear model with each symptom dimension as an independent variable, and one of the behavioral metrics as the dependent variable. Then, the behavioral metrics should be tested for their independence. If the behavioral metrics are correlated, then any discovered relationships should be tested for independence using a partial correlation that removes variance explained by the other behavioral metric.

## Mental Activity is Instantiated by Neural Mechanism

Finally, mental activity is proposed to be instantiated by a network of brain regions.^35^ During the associated mental activity, the network will exhibit a characteristic electrophysiological signal (temporal analysis in EEG) and will recruit activation of component regions (spatial analysis in fMRI). For symptoms of psychiatric illness, the network associated with symptom presentation often includes a control hub in prefrontal cortex (PFC) with control signals emanating from PFC in the low-frequency range (2-12 Hz).^31,36–38^ While research into the neural basis of mental activity spans many spatiotemporal scales from network activity to synaptic mechanisms, the time signature of mental activity is often near the sub-second range, similar to the time signature of low-frequency electrical fluctuations. For example, electrical oscillations in the theta frequency band (4-8 Hz) track with language perception^39^ and the articulation of language exhibits a pronounced theta-frequency band in the speech envelope.^40^ Thus, the strongest neural correlates for mental activity are likely low-frequency electrical activity in networks of brain regions. EEG provides an ideal starting point for discovering temporal signatures of mental activity with fMRI for spatial localization. Of course, techniques with greater spatial or temporal resolution will improved identification, eg, invasive electrocorticography in patients with MDD.^41^

To enable these investigations, several methods for spectral analysis of task-driven EEG might serve in the discovery of novel biomarkers. First, analysis of neural oscillations requires that investigators ensure the presence of a genuine oscillation. Localizing an oscillation during the resting-state can be achieved by performing a linear fit of the power spectrum in logarithmic space, eg, the FOOOF toolbox,^42^ or during task by using a contrast of conditions and demonstrating a band-limited peak in spectral power.^13^ Researchers should be mindful to emphasize the importance of context, spatial origin, and dynamics of that activity, eg, the gamma oscillation in MDD,^43^ as well as the peak frequency of the oscillation, eg, slower alpha frequency in schizophrenia.^44,45^ Functional connectivity analysis using EEG was enabled by the development of the (weighted) phase lag index, in which the confound of volume conduction was addressed by asserting that a genuine functional connection will exhibit a systematic phase lag due to conduction delay.^46,47^ This approach recently revealed that MDD may be characterized by elevated functional connectivity, particularly between the left and right PFC in the alpha frequency band.^31,48–52^

**Figure 3.**
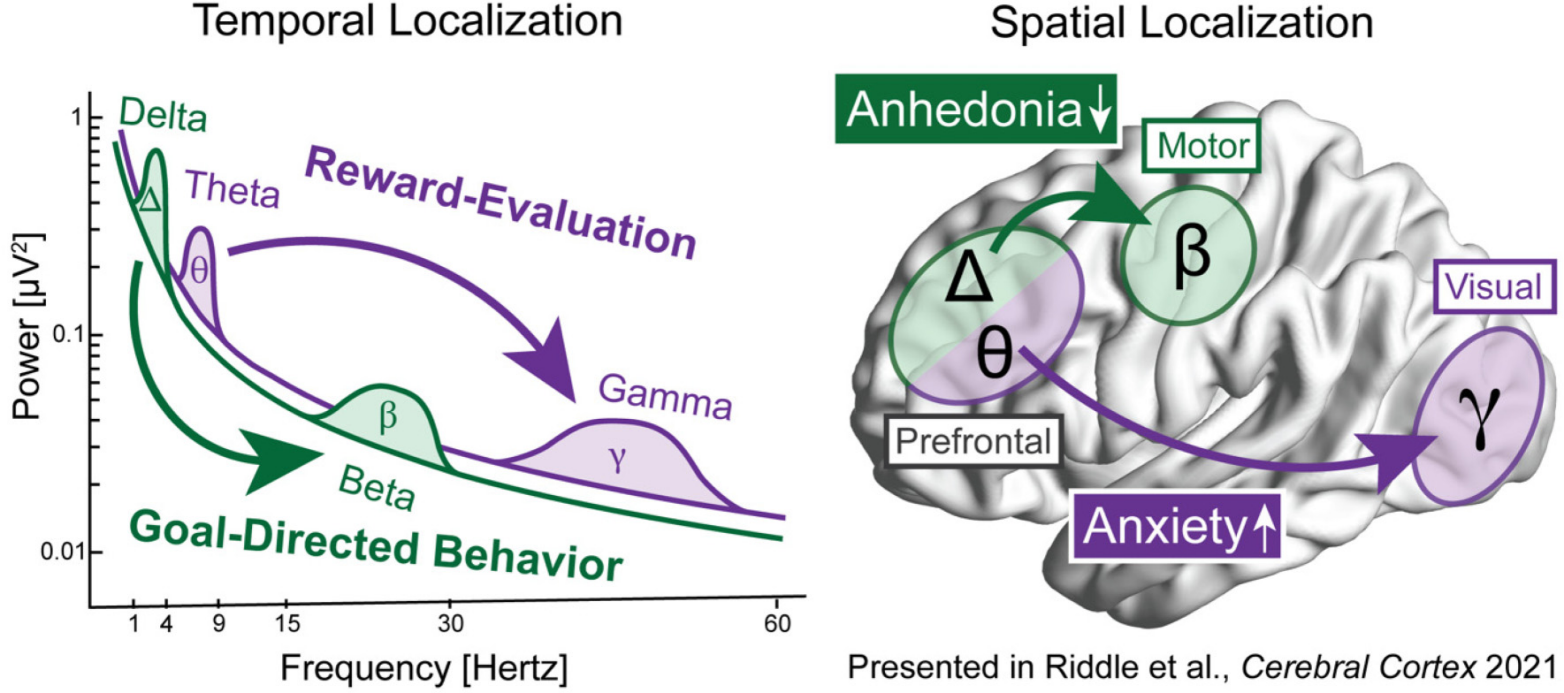
Example neurobehavioral model of symptoms of psychiatric illness. In our recent experiment, we found that distinct temporal (left) and spatial (right) neural mechanisms were related to specific mental activities that were in turn correlated with symptom dimensions in patients with depression. Delta-beta coupling from prefrontal to motor cortex was increased with goal-directed behavior, which was decreased with symptom of anhedonia. Theta-gamma coupling from prefrontal to visual cortex was increased with reward-evaluation, which was increased with symptoms of anxiety.

Finally, cross-frequency coupling between low-frequency control signals in PFC and high-frequency local activity in posterior cortex is proposed to be a fundamental mechanism for cognitive control^53^ that may be altered with psychiatric illness, eg, theta-gamma coupling in schizophrenia.^54,55^ In our recent experiment, we analyzed high-density EEG during a reward-based decision-making task.^13^ We found two distinct modes of top-down control that were correlated with individual differences in behavior. Coupling between delta-frequency (2-4 Hz) activity in prefrontal electrodes and beta-frequency (15-30 Hz) activity in motor electrodes was increased as a function of goal-directed behavior (Figure 3). After isolating prefrontal delta and motor beta activity using Morlet wavelet spectral analysis, we calculated phase-amplitude coupling during the decision period of the task using mean vector length, see Hülsemann et al^56^ for review. Thus, goal-directed behavior likely requires that PFC guides motor-related activity and dysregulation of this activity may underlie symptoms of anhedonia. Critically, our dataset included a range of symptom expression and euthymic control participants with intact delta-beta coupling.

In a similar manner, coupling between theta-frequency (4-8 Hz) activity in prefrontal electrodes and gamma-frequency (30-50 Hz) activity in posterior electrodes was increased as a function of reward-evaluation. Thus, prefrontal control over visual processing is likely increased when incentives need to be appraised and incorporated into decision-making. Anxiety may result in increased appraisal of sensory stimuli from hypervigilant attention to the environment. Intriguingly, a recent study found a similar association between increased prefrontal theta oscillations in anxious depression and reduced delta oscillations with non-anxious depression in response to punishment and reward respectively.^33^ Collectively, these data provide novel biomarkers that may be the target of future intervention. For example, deficient delta-beta coupling in anhedonia could be restored by NIBS.

## Correlation, Causation, and Context

NIBS intervention could be improved by more accurate spatiotemporal targeting and by guiding the mental activity of the patient during intervention. For example, a recent study found that NIBS therapy reduced specific symptoms of depression dependent on the targeted neural circuitry.^15^ When the dorsomedial PFC was targeted, anxiosomatic symptoms were reduced, whereas targeting the dorsolateral PFC reduced anhedonia symptoms. This finding emphasizes the translational importance of dimensional approaches. Future research is required to associate these prefrontal sites with the cross-frequency coupling patterns previously discussed to be related to these symptom dimensions.

It is critical that correlational investigation is followed by causal validation. We recommend that NIBS provides the best avenue for testing the causal relevance of a brain network to a mental activity and symptom expression. For example, rhythmic TMS can be used to target a specific brain region localized from fMRI analysis and to target a specific electrical signal localized from EEG analysis, ie, a single neural oscillation^57,58^ or coupling pattern.^59^ To test for the temporal- and spatial-specificity of the targeted network activity, we recommend that investigators use an arrhythmic pattern of control stimulation,^60–62^ an active control frequency,^60,63,64^ or deliver rhythmic TMS to a site of non-interest.^63,64^ With a spatial and temporal control built into the experimental design, the researchers can provide more compelling evidence that the network activity plays a causal role in generating the mental activity.

In addition, tACS ^65,66^ offers high temporal precision in that the electrical waveform can be customized,^58^ eg, to target cross-frequency coupling neural activity patterns,^67–69^ and network-targeted through diffuse weak electrical stimulation to multiple nodes that comprise the target network.^70–72^ Furthermore, tACS offers similar promise of personalization as TMS and with improved translational relevance as tACS does not require a medical monitor and can be delivered at-home with only minimal safety concerns.^73^

Previous research shows that the impact of NIBS is context-dependent. When the patient is actively generating a known endogenous neural activity pattern, then stimulation targeted to mimic that activity shows greater efficacy.^60,66,74,75^ Despite recent improvements in NIBS treatment protocols, further improvements can be made by driving the underlying brain activity with the associated mental activity and designing NIBS techniques that mimic the endogenous neural activity and carefully target the appropriate brain network. For example, the site in dorsolateral PFC with peak anti-correlation to subgenual cingulate cortex was demonstrated to be the optimal target for TMS in treatment-resistant depression^76^; however, it is unclear what is the neural mechanism for this anti-correlation. The electrophysiological signature is unknown and its identification could improve treatment targeting. Furthermore, it is unknown what is the associated mental activity relevant to treatment response, but goal-directed behavior is consistent with the role of dorsolateral PFC in the frontal-parietal executive control network.

During neurobehavioral model testing, the researcher is encouraged to quantify the impact of stimulation on all aspects of the neurobehavioral model: spatial and temporal activity, task performance, and symptom severity. However, we expect that alleviating symptoms will require multi-day therapeutic interventions.^77–80^ We caution that while the acute effect of stimulation (online) demonstrates the expected increase in the targeted brain activity pattern, chronic forms of stimulation have sometimes been shown to produce the opposite response pattern leading to the theory that chronic stimulation engages homeostatic reset processes.^31,81^

## Future Directions

We forecast that the future of psychiatric intervention will emerge from more comprehensive models for how alterations in mental activity correspond with distinct symptom dimensions. These interventions will be highly personalized to the patient, and will include NIBS as a means of enhancing network activity patterns concurrent with psychotherapy or tasks designed to drive constructive mental activity.^82^ Finally, closed-loop stimulation offers a unique means of continuously monitoring the neural and/or mental activity of the patients and adjusting stimulation in real-time to constructively shift neural activity towards a desired state.^83^
